# Live-Attenuated VEEV Vaccine Delivered by iDNA Using Microneedles Is Immunogenic in Rabbits

**DOI:** 10.3389/fitd.2022.813671

**Published:** 2022-03-18

**Authors:** Irina Tretyakova, Mark Tomai, John Vasilakos, Peter Pushko

**Affiliations:** 1 Medigen, Inc., Frederick, MD, United States; 2 3M Company, St. Paul, MN, United States; 3 Kindeva Drug Delivery, St. Paul, MN, United States

**Keywords:** DNA vaccine, live-attenuated vaccine, iDNA, Venezuelan equine encephalitis virus, VEE, TC83, V4020, microneedle

## Abstract

Effective and simple delivery of DNA vaccines remains a key to successful clinical applications. Previously, we developed a novel class of DNA vaccines, sometimes called iDNA, which encodes the whole live-attenuated vaccine viruses. Compared to a standard DNA vaccine, an iDNA vaccine required a low dose to launch a live-attenuated vaccine *in vitro* or *in vivo*. The goal of this pilot study was to investigate if iDNA vaccine encoding live-attenuated Venezuelan equine encephalitis virus (VEEV) can be efficiently delivered *in vivo* by a microneedle device using a single-dose vaccination with naked iDNA plasmid. For this purpose, we used pMG4020 plasmid encoding live-attenuated V4020 vaccine of VEE virus. The V4020 virus contains structural gene rearrangement, as well as attenuating mutations genetically engineered to prevent reversion mutations. The pMG4020 was administered to experimental rabbits by using a hollow microstructured transdermal system (hMTS) microneedle device. No adverse events to vaccination were noted. Animals that received pMG4020 plasmid have successfully seroconverted, with high plaque reduction neutralization test (PRNT) antibody titers, similar to those observed in animals that received V4020 virus in place of the pMG4020 iDNA plasmid. We conclude that naked iDNA vaccine can be successfully delivered *in vivo* by using a single-dose vaccination with a microneedle device.

## INTRODUCTION

Venezuelan equine encephalitis virus (VEEV) is a mosquito-borne alphavirus (*Togaviridae* family), which causes human disease outbreaks and equine epizootics, mostly in the South, Central, and North America (1, 2). Climate, ecological changes, and international travel increase the risk of VEEV reemergence (3-5). In addition, VEEV is a potential bioterrorism threat (3). Currently, there is no licensed human vaccine for VEEV, and the potential risks of VEEV outbreaks necessitate the development of a safe and effective vaccine (6).

DNA-based vaccines have great potential as a vaccination strategy due to the simplicity of production, genetic and chemical stability, and no requirement for a cold chain. We recently developed an iDNA approach to launch live-attenuated vaccines from DNA plasmids. Live-attenuated vaccines have the advantage of rapidly inducing natural, long-lasting, protective immunity, which makes them the ideal vaccines to contain outbreaks. However, because of the high rates of mutation in RNA viruses (7, 8), there is a concern that live vaccines consist of a heterologous population and can potentially acquire reversion mutations in the process of multiple passages during vaccine production, leading to regeneration of the wild-type, virulent phenotype. For example, safety considerations have hindered Food and Drug Administration (FDA) approval of TC83, a live-attenuated VEEV vaccine developed in the 1960s (9, 10). The TC83 vaccine includes two attenuating mutations, 5′A3 and E2-Arg120. Genetic reversions have been associated with the risk of adverse reactions (11). In contrast to a standard virus vaccine representing a population of viruses, an inherently stable iDNA plasmid represents a genetically defined vaccine, which may provide a safety advantage.

In addition, V4020 VEEV iDNA vaccine was engineered for improved safety, including additional attenuation strategies for preventing reversion mutations (12). The V4020 vaccine was prepared using an iDNA infectious clone that encodes the full-length rearranged genomic RNA downstream from the optimized CMV promoter. A recombinant plasmid pMG4020 encoding the genomic RNA of the V4020 vaccine virus has been confirmed to launch replication of a live-attenuated V4020 vaccine virus *in vitro* and *in vivo* (12, 13). Safety, immunogenicity, and protection of V4020 virus against VEEV challenges were confirmed in BALB/c mice and non-human primates (12, 14). A small dose of iDNA can be used to launch a live-attenuated virus. However, in order to launch V4020 virus *in vivo*, iDNA was previously delivered either using a transfection reagent or by electroporation, similarly to traditional DNA vaccines (12, 13).

In this study, we report that naked iDNA plasmid can be efficiently delivered *in vivo* using a simple, single-dose vaccination using a hollow microstructured transdermal system (hMTS) microneedle technology.

## MATERIALS AND METHODS

### Cell Line, Plasmid, and Virus

**FIGURE 1 ∣:**
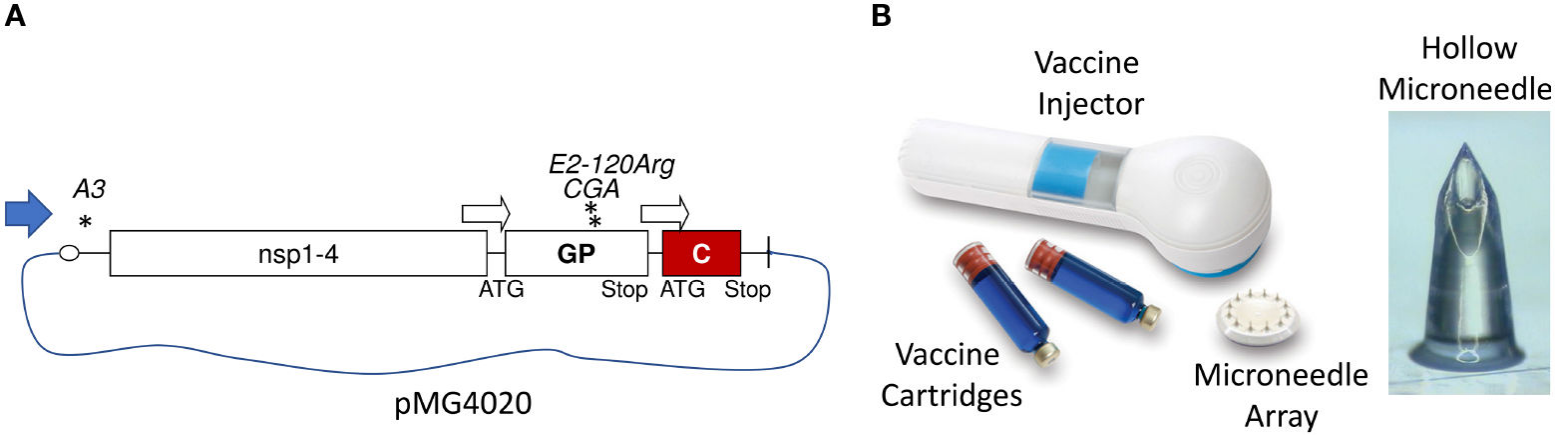
Genetic structure of the pMG4020 plasmid and V4020 VEEV vaccine virus with rearranged genome. **(A)** RNA genome map of V4020 virus. Indicated are 5′ cap, major open reading frames (nsP1-4, GP, C), 26S subgenomic promoters (open arrows), and 3′-Poly **(A)**. Asterisks indicate attenuating mutations. Map is not to scale. **(B)** hollow microstructured transdermal system (hMTS) transdermal hollow microneedle device. Shown are vaccine injector, vaccine cartridges, microneedle array, and enlarged hollow microneedle structure. The array contains 12 microneedles; each microneedle is 1,500 μm in length. For vaccination of rabbits, vaccine cartridges were aseptically pre-loaded with 20 μg of pMG4020 or 10^4^ PFU of V4020 live-attenuated virus. Cartridges were installed into vaccine injector, and vaccine was delivered transdermally to rabbit skin.

The pMG4020 plasmid was described elsewhere (12). Briefly, pMG4020 encoded the full-length, rearranged RNA genome of the V4020 vaccine virus (Figure 1). The pMG4020 plasmid was grown in *Escherichia coli* in the presence of kanamycin and isolated using an endotoxin-free DNA isolation method (Qiagen, Valencia, CA, USA).

Vero cells (African green monkey) from the American Type Culture Collection (CCL-81.4, Manassas, VA, USA) were maintained in a humidified incubator at 37°C and 5% CO_2_ in αMEM supplemented with 10% fetal bovine serum (FBS) and gentamicin sulfate (10 μg/ml) (Life Technologies, Carlsbad, CA, USA).

The V4020 live-attenuated vaccine virus was prepared by transfecting Vero cells with pMG4020. Transfection was done by electroporation essentially as described elsewhere (13). Briefly, Vero cells were transfected with 1 μg of pMG4020 and incubated at 37°C. The growth medium (passage P0) was harvested at 20 h post-transfection. The V4020 P0 virus was used to infect Vero cells at a multiplicity of infection (MOI) of 0.01 to generate the V4020 passage P1 virus in 175-cm^2^ flasks. The P1 vaccine virus was harvested, concentrated 100-fold, partially purified by ultracentrifugation, resuspended in phosphate-buffered saline (PBS), and filter-sterilized using a 0.2-μm filter. The titer of the V4020 P1 was determined by plaque assay and adjusted with PBS to 1 × 10^8^ PFU/ml.

Both pMG4020 plasmid and V4020 vaccine virus were confirmed for the ability to express VEEV antigens in Vero cells by immunofluorescence assay (IFA) and Western blotting. Plaque morphology was evaluated in Vero cells by plaque assay as described elsewhere (12, 13).

### Microneedle Devices

Microstructured transdermal system (MTS) from 3M (Kindeva) Drug Delivery (Saint Paul, MN, USA) provides needle-free intradermal administration of small molecules and macromolecules. For vaccinations, we used a hMTS platform designed for the delivery of non-viscous liquid formulations including vaccines. The hMTS devices were a gift from 3M Corporation. These devices are prepared using medical-grade polymer and designed for fast, high-efficiency drug delivery using simple and accurate drug administration.

The cartridges for hMTS devices were aseptically filled with 0.5 ml of vaccines (either pMG4020 iDNA or V4020 virus) according to the manufacturer’s instructions. Care was taken to avoid air bubbles in the prefilled cartridges. Three hMTS devices were filled with pMG4020, while one hMTS device was filled with control V4020 live virus vaccine. The filled cartridges were crimped to properly seal the cartridge.

Immediately before vaccine application to animals, the vaccine-filled cartridges were inserted into the injector to align with the spring-powered vaccine delivery plunger mechanism. The hMTS injector contained an array of twelve preinstalled hollow microneedles to administer the vaccine to the injection site (Figure 1).

### Immunizations of Rabbits

Animal research involving experimental rabbits was done according to the approved institutional protocol (Noble Life Sciences No. 493). New Zealand white rabbits were purchased from Charles River. Animals were divided into two groups, three per group. Rabbits (2.5–3 kg, adult, female) were anesthetized prior to vaccinations. One group of three animals was vaccinated using pMG4020 plasmid encoding the V4020 virus. Another group was vaccinated using the V4020 virus. Either vaccine (pMG4020 iDNA plasmid or V4020 virus) was administered using hMTS microneedle devices according to the manufacturer’s instructions. To minimize the number of animals due to ethical and animal welfare reasons, one control rabbit was injected with V4020 vaccine virus using the hMTS device, and one control rabbit was injected with V4020 vaccine virus using a standard syringe needle *via* subcutaneous (SC) route of administration.

The vaccine was administered on day 0. Animals were vaccinated with 0.5 ml of pMG4020 iDNA (20 μg) or V4020 virus vaccine (10^4^ PFU) using hMTS microneedle devices or with 0.5 ml (10^4^ PFU) of V4020 vaccine SC using a standard syringe in the right leg at a titer of 2 × 10^4^ PFU/ml. The human dose of the TC83 vaccine is 10^5^ PFU; therefore, 10^4^ PFU dose was chosen for rabbits considering the difference in body weight.

The animals were placed under general anesthesia using a combination of ketamine (50 mg/kg) and xylazine (10 mg/kg), and the site of injection was cleanly shaved using clippers followed by a razor prior to application of the device and injection. The injection site skin was stretched and secured onto a solid support bar. The device was attached to the skin *via* a self-adhesive surface, and the injection button on the device was depressed to deliver the pre-loaded vaccine cargo from the cartridge through the microneedles. As a control, one animal was dosed SC with 0.5 ml of the attenuated VEE vaccine strain V4020 at the same amount and concentration as those in the hMTS microneedle array device.

After vaccinations, animals were observed daily. Blood samples were taken on days 0, 7, and 21. On days 0 (vaccination), 7, and 21, rabbits were bled *via* the auricular artery for assessment of viremia (day 7) and the humoral response (neutralizing antibodies and IFA) to the vaccines. In a previous study, viremia was detectable up to 8 days in cotton rats after SC inoculation with 3 log10 PFU of VEEV (15).

### Evaluation of Viremia

After vaccination, viremia was evaluated on day 7 post-vaccination by either direct plaque assay on Vero cell Monolayers or virus amplification in Vero cells.

Briefly, 0.1 ml of serum was used for direct plaque assay. Plaques were visualized with neutral red. For virus amplification, 1 ml of serum was incubated in 75-cm^2^ flasks, and cell monolayers were examined daily for cytopathic effect (CPE), while cell supernatant was examined for replicating virus using plaque assay.

### Evaluation of Neutralizing Antibody

Neutralizing antibodies were determined by plaque reduction neutralization tests (PRNT_80_ and PRNT_50_) in duplicate in Vero cell monolayers. For PRNT, sera were heat inactivated, and VEEV V4020 was mixed with serum dilutions before plaque assay. Dilution of serum required to cause 80% and 50% reduction of plaques was determined.

As controls, pre-bleed sera (day 0) were used to compare PRNT titers of sera from days 7 and 21 post-vaccination to PRNT titers of pre-bleed. Statistical analysis was performed using the mean logarithm of titer and Student’s t-test. Statistical analysis for potential differences between groups was not performed due to small group sizes. Cohorts of 3 animals yield adequate power to show vaccine immunogenicity (p < 0.05).

To confirm seroconversion, indirect IFA was used. Briefly, Vero cell monolayers were infected in chamber slides with V4020 virus at MOI = 0.1 for 24 h. Monolayers were fixed with cold acetone and probed with rabbit antiserum at 1:25 dilution, followed by secondary fluorescein isothiocyanate (FITC)-labeled anti-rabbit IgG (H+L) at 1:25 dilution. Monolayers were covered with a mounting medium containing propidium iodide (Vector Labs, Burlingame, CA, USA). V4020-specific rabbit serum antibodies were detected by observing fluorescent foci of V4020-infected cells.

## RESULTS

### VEEV Vaccines pMG4020 and V4020

The genome of the V4020 VEEV virus with gene rearrangement and attenuating mutations is schematically shown in Figure 1. In the V4020 RNA, the capsid (C) gene was expressed using the duplicated subgenomic 26S promoter downstream from the glycoprotein (GP) genes, resulting in a gene rearrangement. V4020 also maintained both attenuating mutations 5′A3 (in the 5′ untranslated region) and E2-120Arg (in the E2 gene) derived from the TC83 sequence. However, the translational codon AGA encoding attenuating mutation E2-120Arg in the TC83 virus (16) was changed in V4020 to a synonymous codon CGA, which requires at least two mutations to revert to the wild-type VEEV E2-120Thr codon ACA (12, 14). V4020 was isolated from *E. coli* using the endotoxin-free method.

V4020 vaccine virus was prepared by transfecting Vero cells with pMG4020 plasmid encoding the full-length infectious RNA genome of rearranged V4020 vaccine virus (Figure 1) as described in the Materials and Methods. The titer of the resulting V4020 vaccine virus, 4 × 10^8^ PFU/ml, was determined by standard plaque assay in Vero cell monolayers confirming synthesis of vaccine virus from the plasmid *in vitro*. Both iDNA plasmid and V4020 live virus vaccines were filter-sterilized before aseptically loading into hMTS devices or standard syringes.

### Vaccination Using Hollow Microstructured Transdermal System Hollow Microneedle Devices

Rabbits are often used as a non-rodent animal model for the evaluation of DNA vaccines (17). Vaccination of experimental rabbits was done using hMTS hollow microneedle devices designed for the delivery of liquid formulations, representing the first application of hMTS devices in combination with iDNA (Figure 1). These devices are designed to accurately and consistently administer drugs into the dermis. The hMTS devices were preloaded with 0.5 ml of each vaccine containing either 20 μg of pMG402 iDNA plasmid or 10^4^ PFU of live V4020 vaccine virus (control). Another control animal received a 0.5-ml dose of 10^4^ PFU of live V4020 vaccine virus *via* standard SC inoculation by a syringe needle. All vaccines were formulated in PBS and sterile-filtered. After administration, the remaining vaccines were quantitated to confirm the quantity and titer of vaccines administered to experimental animals.

### Immunogenicity of pMG4020 iDNA and V4020 Virus Vaccines in Rabbits

**FIGURE 2 ∣:**
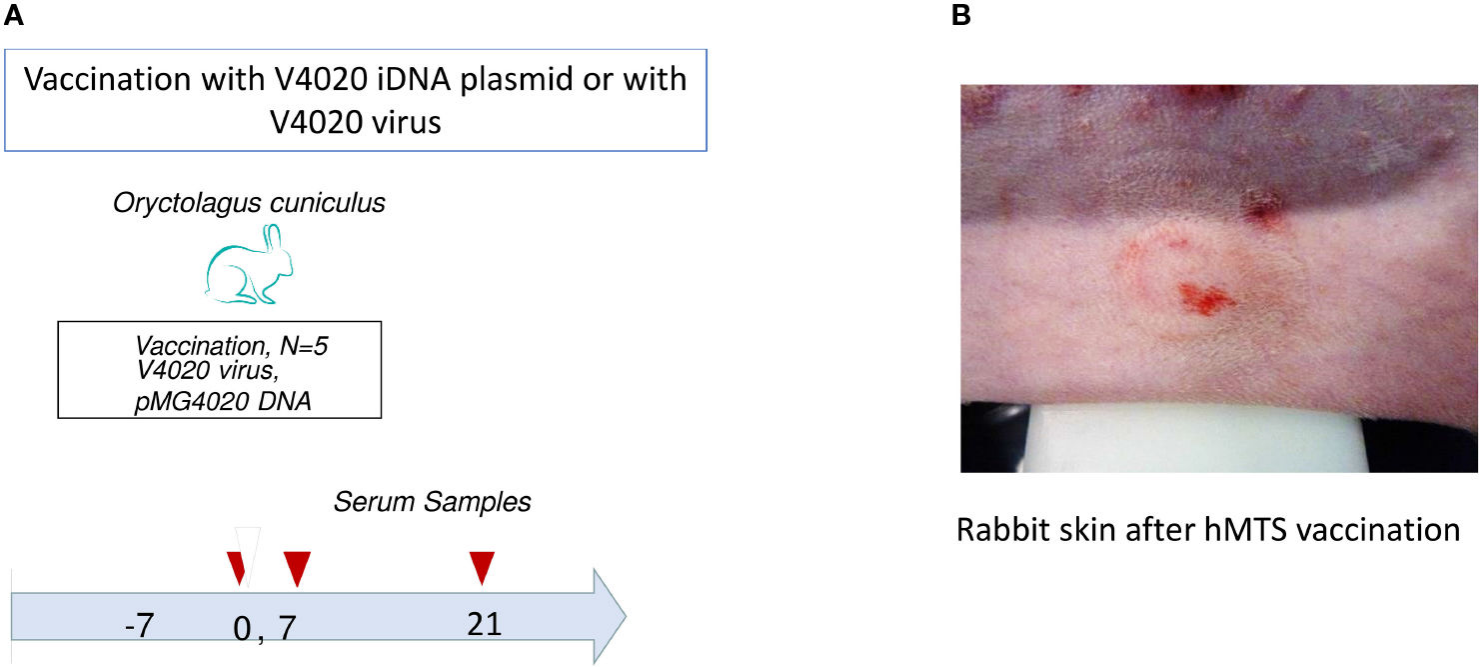
**(A)** Schematic diagram of vaccination of rabbits (*Oryctolagus cuniculus*) with the pMG4020 or V4020 vaccine. The study timeline is shown from day −7 before vaccination through day 38 after vaccination. Vaccinations were performed on day 0 (open triangle). Blood draws are indicated with filled triangles. Rabbits were vaccinated with 20 μg of pMG4020 transdermally using hollow microstructured transdermal system (hMTS) hollow microneedle devices, or with 10^4^ PFU V4020 live-attenuated using hMTS devices, or with 10^4^ PFU of V4020 virus using syringe needle SC as described in the Materials and Methods. On indicated days, blood was drawn for serology. **(B)** Rabbit skin after vaccination with pMG4020 and hMTS microneedle device.

The proof-of-concept study in rabbits was performed to test the vaccine administration using hMTS microneedle devices. The hMTS devices were loaded with vaccines as described in the Materials and Methods. Rabbits were vaccinated on day 0 SC with a single dose of either 20 μg of pMG4020 or 10^4^ PFU of V4020 vaccine virus (Figure 2 and Table 1).

All animals were closely monitored after vaccination signs of illness from either the pMG4020 or from the V4020 vaccine virus including their appearance, behavioral changes, and food and water consumption. All animals assigned to the study survived for the duration of the study. There were no apparent adverse reactions to vaccination. No abnormal behavior was noted during the entire period of observation. Serum samples collected on day 7 post-vaccination were examined for virus neutralization and viremia. Viremia was not detectable at day 7 post-vaccination either by direct plaque assay or by amplification in Vero cell monolayers, suggesting that there is no detectable viremia in rabbits on day 7 after vaccination.

**FIGURE 3 ∣:**
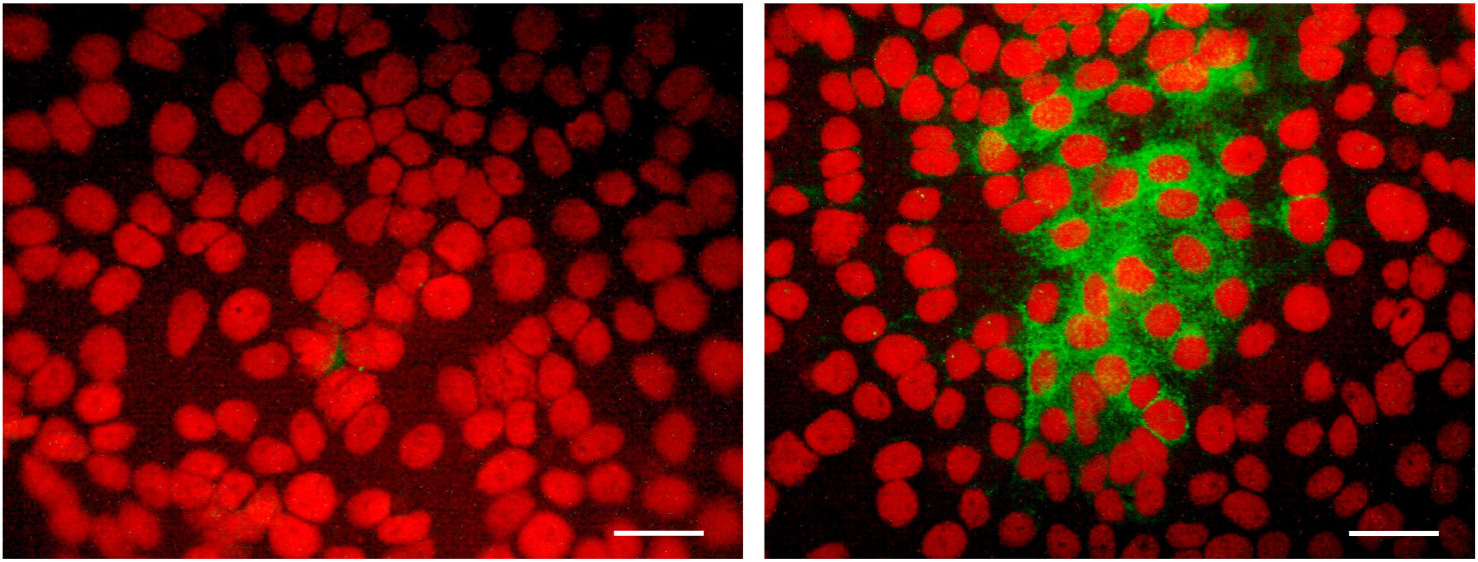
Detection of V4020-specific antibody by immunofluorescence assay (IFA) in Vero cell monolayers infected with V4020 virus at multiplicity of infection (MOI) = 0.1. Serum was collected from pMG4020-vaccinated rabbit on day 0 (left panel) and day 21 (right panel) and used as primary antibody to detect foci of V4020 virus in Vero cell monolayers. Secondary antibody was fluorescein isothiocyanate (FITC)-labeled goat anti-rabbit IgG (H+L). Mounting medium containing propidium iodide counterstain (Vector Labs, Burlingame, CA, USA) was used to visualize nuclei of the cells in red fluorescence. Positive green fluorescence indicates detection of VEEV antigen using serum antibody (day 21) from vaccinated rabbits. Bar, 50 μM.

On day 21, all three vaccinated rabbits in the iDNA-vaccinated group seroconverted on day 21, as shown by PRNT_80_ and PRNT_50_. The PRNT_80_ titers varied from 40 to 640 (Table 1). The two-sided t-test was statistically significant. One-sided t-test also showed statistical significance p < 0.05 (p = 0.012). The PRNT_80_ titer of 640 was also determined in animals vaccinated *via* hMTS microneedle devices with V4020 live vaccine. The PRNT_50_ titers also showed seroconversion on day 21 in either pMG4020- or V4020-vaccinated animals (Table 1). One animal in the pMG4020-vaccinated group showed slightly lower PRNT_80_ and PRNT_50_ titers, at 40 and 160, respectively. To confirm seroconversion in this animal, serum was probed by IFA using Vero cell monolayers infected with the V4020 virus at MOI 0.1. Positive fluorescent foci were detected using rabbit serum collected on day 21 but not on day 0 (Figure 3), thus confirming seroconversion, also detected by PRNT_80_ and PRNT_50_ (Table 1). Surprisingly, the animal that received a single-dose SC vaccination *via* standard syringe inoculation did not seroconvert, suggesting a potential technical issue (Table 1).

Taken together, these results demonstrated that both pMG4020 naked DNA and V4020 virus vaccinations are safe and immunogenic *via* hMTS hollow microneedle devices. Additional studies are warranted to confirm this proof-of-concept observation.

## DISCUSSION

Efficient vaccination with DNA vaccines remains among the most important goals of current vaccinology (18-20). Although shown safe and effective in animal models, standard DNA vaccines demonstrate limited success in humans and sometimes require large doses, multiple booster vaccinations, advanced adjuvants, novel formulations, and/or complex delivery equipment including *in vivo* electroporation. It should be also noted that traditional DNA vaccines encode mRNA to express subunit vaccines. Subunit vaccines themselves often have low immunogenicity and require adjuvants for improved immunogenicity and efficacy (21).

To overcome the hurdle of effective implementation of DNA-based vaccines, in this pilot feasibility study, we combined two innovative technologies, iDNA and microneedles, to deliver DNA-based vaccines *in vivo*.

Firstly, we used recently developed “immunization DNA” (iDNA) that encodes the full-length genomic RNA of the live attenuated VEEV vaccine. Thus, unlike standard DNA vaccines, an iDNA encodes the whole live-attenuated virus. Only a small dose of iDNA is required to launch a live-attenuated vaccine, which then self-replicates and induces efficient immune response and protection as demonstrated in animal models (12-14, 22).

Secondly, we configured hMTS hollow microneedle platform for delivery of liquid formulations of iDNA and virus vaccines *in vivo*. These devices are designed for easy, comfortable self-application, eliminating standard syringe needles. The hMTS can be used for fast, high volume, efficient pain-free delivery of liquid formulations including vaccines (23). Microneedles are easy to dispose of, and there is no potential for accidental needle exposure. They are expected to generate pharmacokinetic profiles similar to SC administration with some drugs. Potentially, hMTS combine the comfort of a patch with the efficiency of a syringe and can be used to minimize transitional studies from approved formulations currently administered by other injection routes. In the previous human clinical studies, minimal discomfort was associated with the application of hMTS arrays, and the hMTS infusion was well-tolerated (24). The hMTS structures are made from medical-grade, class VI materials, and a cap protects the microneedle array. Upon removal of the cap, the patient simply adheres to the single-use delivery system to the skin of his or her thigh or abdomen and presses down to begin dosing of liquid formulations of 0.5 to 2 ml.

Here we used hMTS devices to deliver PBS-formulated iDNA vaccine for VEEV to rabbits for proof-of-concept immunogenicity study. VEEV is capable of causing explosive outbreaks and can be spread by many mosquito vectors including *Culex, Mansonia, Psorophora*, and *Aedes* species (5, 25-27). Live-attenuated vaccines are among the most cost-effective and broadly used public health measures representing approximately half of all FDA-licensed vaccines. In recent years, live vaccines Zostavax, FluMist, Rotarix, and others have been approved for human use, demonstrating that live vaccines can be configured to meet FDA safety standards (28, 29). The TC83 live-attenuated vaccine for VEEV was developed decades ago by classic virology using multiple passages in tissue culture to select attenuating mutations (9, 16). Previously, we have reported the rearranged V4020 vaccine (12). Rearranged genomes lead to attenuation in many viruses including VEEV, which are resistant to reversions because many independent mutations are required to restore the wild-type virus sequence (29-32). The V4020 vaccine includes both genomic rearrangements and genetically stabilized attenuating mutations derived from the TC83 vaccine. In a previous study in BALB/c mice, V4020 and TC83 had comparable immunogenicity and protective efficacy, while V4020 had safety advantages (12, 33). We also showed that V4020 derived from pMG4020 iDNA is immunogenic and efficacious in cynomolgus macaques against aerosol challenge with pathogenic VEEV (14). Recent examples of other promising VEEV vaccines also included standard DNA vaccines expressing VEEV antigens (34-36).

When rapid containment of outbreaks is needed, live-attenuated vaccines have advantages because they induce rapid and strong immunity with a single dose. Cost considerations are important when a large number of doses are needed for vaccination, for example, in endemic countries (37, 38). The pMG4020 iDNA is a novel way to deliver live-attenuated VEEV vaccine *in vivo via* DNA immunization. It has been shown that iDNA is launching live-attenuated viruses *in vitro* or *in vivo* (12, 13, 22, 39). V4020 can be either prepared *in vitro* or used for vaccination as a live-attenuated vaccine, or it can be synthesized in tissues *in vivo* using vaccination with pMG4020 plasmid encoding RNA genome of V4020. In the iDNA infectious clone technology, the full-length RNA is transcribed in the nucleus from the plasmid and transported into the cytoplasm. We also used iDNA infectious clone technology to prepare experimental vaccines for the chikungunya alphavirus (39), as well as for yellow fever and Japanese encephalitis flaviviruses (40, 41). In this study, we provide proof-of-concept feasibility data that iDNA vaccines such as the pMG4020 VEEV vaccine can be potentially delivered by a microneedle technology as naked DNA to launch the live-attenuated vaccine *in vivo*. Potentially, multiple types of microneedle and other delivery technologies can be utilized for efficient iDNA vaccination. More studies are planned, including the length of vaccine production from the iDNA plasmid, innate and cellular immune responses, and ELISA antibody titers and assessing chemistry of serum (BMP + AST/ALT + muscle enzymes such as CK/aldolase) to evaluate potential reactogenicity of vaccination. The DNA format has multiple advantages, such as longer shelf life in warm climates (22) and genetic stability, while the ability to launch live attenuated vaccine provides the iDNA with the ability to induce immune response and protection with a single vaccination, which can be especially important in epidemic scenarios.

## Figures and Tables

**TABLE 1 ∣:** Immunogenicity of pMG4020 and V4020 vaccines in rabbits.

Vaccine, route	Day 0		Day 7		Day 21	
	Vaccine dose	PRNT_50/80_	Viremia direct, PFU/ml	Viremia, by amplification, PFU/ml	PRNT_80_	PRNT_50_
1. V4020, SC	10^4^ PFU	< 10	< 10	< 1	< 10	< 10
2. V4020, TD	10^4^ PFU	< 10	< 10	< 1	640	5,120
3. pMG4020, TD	20 μg	< 10	< 10	< 1	320	1,280
4. pMG4020, TD	20 μg	< 10	< 10	< 1	40	160
5. pMG4020, TD	20 μg	< 10	< 10	< 1	640	1,280

Pre-bleed sera (day 0) were used to compare PRNT titers of sera from days 7 and 21 post-vaccination to PRNT titers of pre-bleed. Statistical analysis was performed using mean logarithm of titer (day 21) and one-sided Student’s t-test.

V4020, live-attenuated virus; pMG4020, iDNA plasmid; SC, subcutaneously using syringe; TD, transdermally using 3M (Kindeva) hMTS devices; PRNT, plaque reduction neutralization test.

## Data Availability

The original contributions presented in the study are included in the article/supplementary material. Further inquiries can be directed to the corresponding author.
